# Variations in TcdB Activity and the Hypervirulence of Emerging Strains of *Clostridium difficile*

**DOI:** 10.1371/journal.ppat.1001061

**Published:** 2010-08-19

**Authors:** Jordi M. Lanis, Soumitra Barua, Jimmy D. Ballard

**Affiliations:** Department of Microbiology and Immunology, The University of Oklahoma Health Sciences Center, Oklahoma City, Oklahoma, United States of America; Harvard Medical School, United States of America

## Abstract

Hypervirulent strains of *Clostridium difficile* have emerged over the past decade, increasing the morbidity and mortality of patients infected by this opportunistic pathogen. Recent work suggested the major *C. difficile* virulence factor, TcdB, from hypervirulent strains (TcdB_HV_) was more cytotoxic in vitro than TcdB from historical strains (TcdB_HIST_). The current study investigated the in vivo impact of altered TcdB tropism, and the underlying mechanism responsible for the differences in activity between the two forms of this toxin. A combination of protein sequence analyses, in vivo studies using a *Danio rerio* model system, and cell entry combined with fluorescence assays were used to define the critical differences between TcdB_HV_ and TcdB_HIST_. Sequence analysis found that TcdB was the most variable protein expressed from the pathogenicity locus of *C. difficile*. In line with these sequence differences, the in vivo effects of TcdB_HV_ were found to be substantially broader and more pronounced than those caused by TcdB_HIST_. The increased toxicity of TcdB_HV_ was related to the toxin's ability to enter cells more rapidly and at an earlier stage in endocytosis than TcdB_HIST_. The underlying biochemical mechanism for more rapid cell entry was identified in experiments demonstrating that TcdB_HV_ undergoes acid-induced conformational changes at a pH much higher than that of TcdB_HIST_. Such pH-related conformational changes are known to be the inciting step in membrane insertion and translocation for TcdB. These data provide insight into a critical change in TcdB activity that contributes to the emerging hypervirulence of *C. difficile*.

## Author Summary

*Clostridium difficile* is a spore-forming bacterium that contaminates hospitals and infects patients undergoing antibiotic therapy. *C. difficile* is now the leading cause of hospital-acquired diarrhea in developed countries. Most concerning has been the recent increase in mortality of *C. difficile* patients due to the emergence of a hypervirulent strain of this pathogen. Results from the current study suggest this change in disease severity may be due to new strains producing a variant form of *C. difficile's* major virulence factor, TcdB. The findings indicate TcdB from hypervirulent strains targets a much broader range of cells in vivo and is able to translocate into target cells more quickly than TcdB from historical strains of *C. difficile*. The more rapid cell entry by TcdB from hypervirulent *C. difficile* appears to be due to the toxin's capacity to undergo conformational changes necessary for membrane translocation at a higher pH than TcdB from historical strains. To date, very little has been learned about the underlying reasons for the increased virulence of emerging *C. difficile* strains. These findings provide insight into this problem and suggest variations in TcdB activity could be an important contributing factor to the hypervirulence of emerging strains of *C. difficile*.

## Introduction

*Clostridium difficile* is a gram-positive, spore-forming anaerobe, first described by Hall and O'Toole over 75 years ago [1]; however, the organism was not associated with human disease until 1978 [2], [3]. Over the past three decades *C. difficile* has become a major nosocomial pathogen and is the leading cause of diarrhea in hospitalized patients [4]. *C. difficile* associated disease (CDAD) is routinely treated by supportive therapy and regimens of vancomycin and metronidazole, but treatment of CDAD has become more difficult due to the emergence of hypervirulent (NAP1/BI/027) strains of *C. difficile* [5], [6], [7]. Elucidating the major differences between historical strains of *C. difficile* and the NAP1/BI/027-related strains of *C. difficile* is critical to understanding how this serious human pathogen continues to emerge.

The phenotypes of hypervirulent and historical strains of *C. difficile* are different [7], [8], [9]. *C. difficile* NAP1/BI/027 produces more toxin and sporulates with higher efficiency than historical strains [6], [7], [8], [9], [10]. NAP1/BI/027 strains also produce a binary toxin, CDT, which is thought to enhance colonization of *C. difficile* by triggering the formation of microtubule protrusions on cells of the gastrointestinal epithelium [11], [12], [13]. Finally, *C. difficile* NAP1/BI/027 strains are resistant to fluoroquinolones due to mutations in DNA gyrase genes [7], [14], [15], [16]. The extent to which one or more of these differences between the two strains contributes to hypervirulence has not been determined.

Recent work from Stabler and colleagues identified several genetic variations between epidemic and historical strains of *C. difficile* [17]. For example, the historical *C. difficile* strain, 630, was found to contain 505 unique coding sequences compared to hypervirulent strains. This analysis also identified differences in flagellar genes, metabolic genes, phage islands, and transcriptional regulators. Of interest to our work was the finding that TcdB from *C. difficile* hypervirulent strains had a greater cytopathic effect on a variety of cell types than TcdB isolated from a *C. difficile* historical strain. The steps in cellular intoxication that account for these differences in TcdB activity, and whether in vivo tropism varies between the historical and hypervirulent TcdB have not been reported.

TcdB (∼269 kDa) is a 2366 residue single polypeptide toxin encoded on a *C. difficile* pathogenicity locus (PaLoc) that also includes genes for two regulators (TcdC and TcdR) of toxin expression, a putative holin (TcdE), and TcdA [18], [19]. TcdB has at least four functional domains that contribute to cell entry and glucosylation of small-GTPases within the cytosol of the cell [20]. TcdB's glucosyltransferase domain is included in the first 516 residues of the toxin, which also includes a conserved DXD motif (Asp286/Asp288) and Trp102, which form a complex with Mn^2+^ and UDP-Glucose [21], [22], [23], [24], [25]. A substrate recognition domain is located between residues 365–516 [26]. The cysteine protease domain at residues 544–955 is necessary for autoproteolytic activity and delivery of the enzymatic domain into the cytosol [27], [28], [29]. A putative membrane-spanning domain resides between residues 956–1128, yet whether this domain is required for intoxication is not known. Finally, the fourth functional domain of TcdB is located within the carboxy-terminal region of the toxin, and is predicted to interact with receptors on target cells [30], [31], [32], [33].

Sequence variations in one or more of the functional domains of TcdB could account for the differences in cytotoxicity between historical and hypervirulent isolates. In the current work we test this hypothesis and demonstrate that TcdB from hypervirulent strains exhibits broader tropism in vivo. We also demonstrate TcdB from hypervirulent *C. difficile* undergoes hydrophobic conformational changes at a higher pH than toxin from the historical strain, and this correlates with more rapid cell entry. These findings provide insight into a possible mechanism through which hypervirulent *C. difficile* causes more severe illness than historical strains of this organism.

## Results

### Sequence comparison of the functional domains of TcdB from a historical strain (TcdB_HIST_) and TcdB from a hypervirulent strain (TcdB_HV_)

The carboxy-terminal sequence of TcdB varies between isolates of *C. difficile,* including hypervirulent and historical strains [17], [34]. Yet, whether sequence variations are more extensive in TcdB compared to other genes in the PaLoc or if the sequences outside of the carboxy-terminal domain of TcdB also varied among different strains of C. *difficile* has not been reported.

We compared the sequences of proteins encoded within the PaLoc of *C. difficile* 630 (a non-NAP1/BI/027 strain) and *C. difficile* R20291 (a 027 strain). The sequence of TcdR, a positive regulator of toxin expression was found to be 100% identical between the two strains of *C. difficile*. TcdE, the putative holin encoded in the middle of the PaLoc exhibited 99% identity and 100% similarity between the two strains of *C. difficile*. The enterotoxin, TcdA, exhibited 98% identity and 99% similarity between the two strains. The gene encoding TcdC from the hypervirulent strain encodes a stop codon and contains a deletion, which made it difficult to precisely compare this protein in the two strains. However, at the DNA level the gene was 95% homologous in the intact coding regions of *tcdC*. In contrast to these almost exact identities of TcdR, TcdE, and TcdA from the two strains, the amino-acid sequence of TcdB from the two strains was found to have the most variation with 92% identity and 96% similarity.

**Figure 1:**
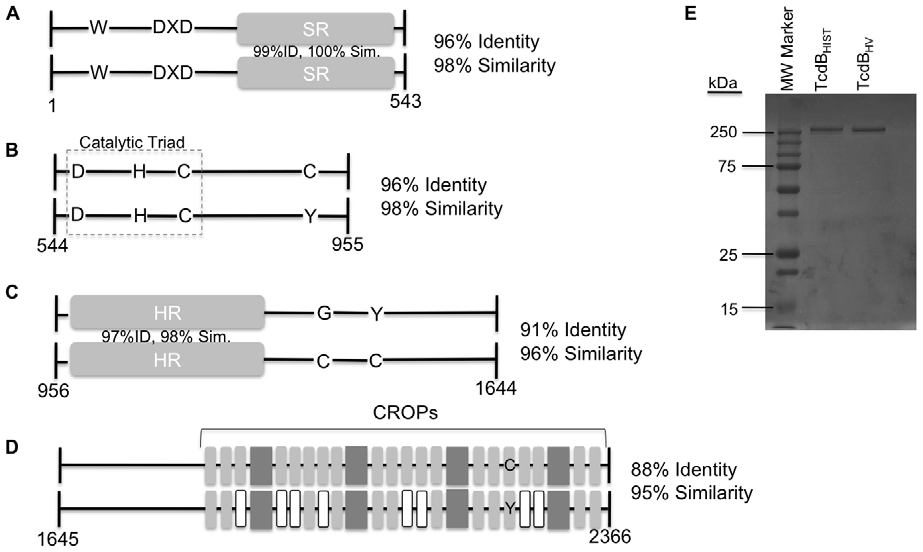
Representation of sequence variation between TcdB_HIST_ and TcdB_HV_. The illustration depicts TcdB_HIST_ (top) and TcdB_HV_ (bottom) divided into functional domains: glucosyltransferase (A), cysteine protease (B), translocation (C), and receptor binding (D). (A) Trp 102 and the DXD motif of the glucosyltransferase domain are conserved between TcdB_HIST_ and TcdB_HV_. The amino acids making up the substrate recognition region (SR) show 99% similarity between the strains, and the overall amino acid identity of the domain is 96%. (B) The catalytic triad of the cysteine protease domain remains unchanged between TcdB_HIST_ and TcdB_HV_, and the overall identity of the domain is 96%. TcdB_HIST_ contains a cysteine at residue 870, while TcdB_HV_ contains a tyrosine at residue 870. (C) Amino acid identity of the translocation domain is 91%, with a 97% sequence identity occurring in the hydrophobic region (HR). TcdB_HV_ contains two cysteines in this domain, which are not found in this region of TcdB_HIST_. (D) TcdB_HIST_ and TcdB_HV_ share an identity of 88% in the putative receptor binding domain. Gray boxes symbolize the CROP (clostridial repetitive oligopeptide) regions, 4 large repeats and 18 small repeats. White boxes indicate TcdB_HV_ CROPs that have less than 80% similarity to TcdB_HIST_. (E) Coomassie stained SDS-PAGE analysis of 1 µg of each TcdB_HIST_ and TcdB_HV_.

We next compared the functional regions of TcdB_HIST_ and TcdB_HV_ (Fig. 1). The enzymatic region of TcdB (encompassing residues 1–543) was found to be 96% identical and 98% similar between the two strains of *C. difficile*. Residues critical for catalytic activity, W102 and the DXD motif, did not vary between the two forms of TcdB (Fig. 1A). The substrate specificity domain of TcdB (residues 365 to 516) [26] exhibited 99% identity and 100% similarity (Fig. 1A). The autoproteolytic region (residues 544 to 955) was found to contain 96% identity and 98% similarity. Moreover, the reported catalytic triad (D587, H653, and C698) was conserved between the two forms of TcdB. Interestingly however, the analysis found a rearrangement of a second cysteine residue in this region of TcdB. TcdB_HIST_ contains a cysteine at residue 870, but this residue is a tyrosine in TcdB_HV_ (Fig. 1B). Conversely, TcdB_HV_ has a cysteine residue at 1477, but this was found to be a glycine residue in TcdB_HIST_. The third putative functional domain of TcdB is between residues 956 and 1644, and encodes a hydrophobic region thought to mediate membrane insertion. Comparison of this region found 91% identity and 96% similarity (Fig. 1C).

In line with earlier reports [17], [34] the carboxy-terminal region, encompassing residues 1645 to 2366, exhibited the highest degree of sequence variation in the toxin. The carboxy-terminal region showed 88% identity and 95% similarity between the two forms of TcdB. The number of CROP regions is identical, with TcdB_HIST_ and TcdB_HV_ containing 24 regions based on the YF consensus motif [30], [32], [35], [36]. However, eight of these regions in TcdB_HV_ were found to exhibit less than 80% sequence identity to TcdB_HIST_ (Fig. 1D).

Fig. 1E shows an SDS-PAGE analysis of TcdB_HIST_ and TcdB_HV_ purified from wild-type strains of *C. difficile* as described in the materials and methods. Both forms of the toxin were obtained at greater than 95% purity based on minimal detection of contaminating proteins.

### In vivo assessment of TcdB_HIST_ and TcdB_HV_

We next used a zebrafish model to compare the in vivo effects of the two forms of this toxin. Our group has previously utilized the zebrafish embryo as a model to examine the effects of TcdB_HIST_ in real time, and found that this toxin had potent cardiotoxic effects [37]. The zebrafish provides a distinct advantage for the purpose of examining tissue damage and tropism because it is possible to visualize these events directly with this model.

**Figure 2:**
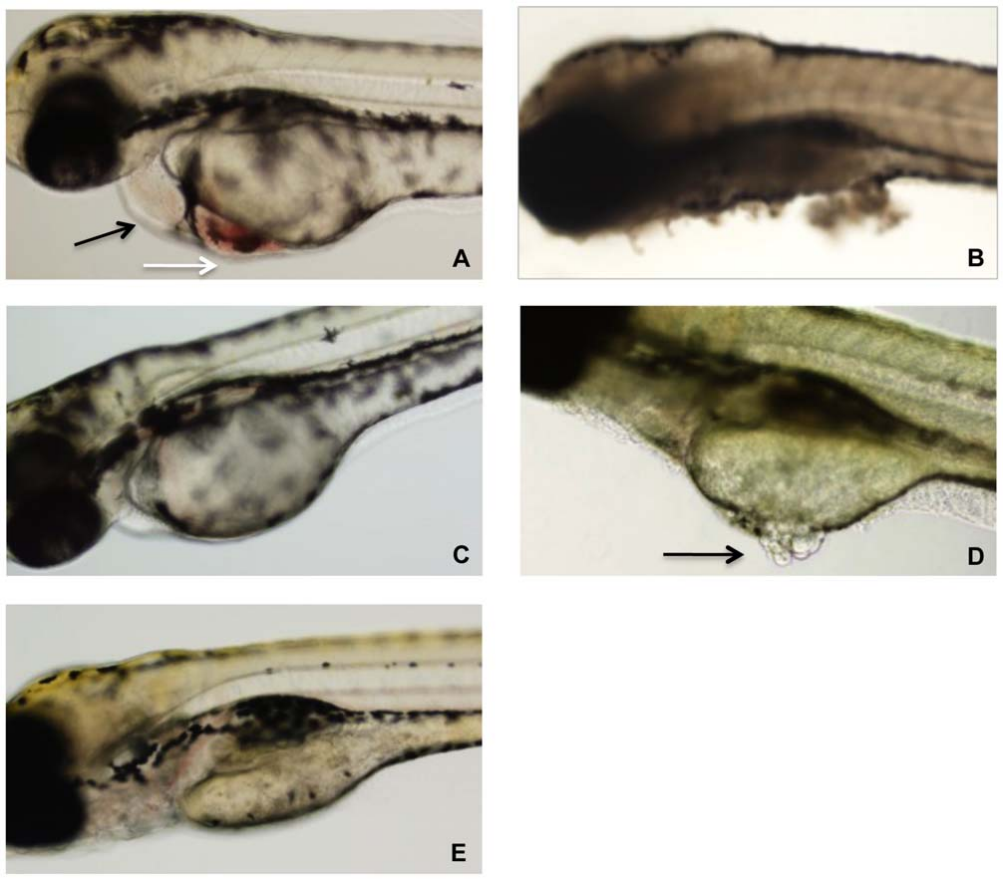
Representative photographs of zebrafish after 24 h exposure to TcdB. (A) Zebrafish after exposure to 10 nM TcdB_HIST_. Cardiac damage is evident by pericardial edema (black arrow) and blood accumulation (white arrow). (B) Exposure to 10 nM TcdB_HV_ causes tissue necrosis and death of the zebrafish. (C) Zebrafish treated with 1 nM TcdB_HIST_ appear normal, with little to no edema. (D) Zebrafish after exposure to 1 nM TcdB_HV_. Arrow indicates damage to the yolk sac, visualized by tissue discoloration and necrosis. (E) Untreated control.

Zebrafish embryos were arrayed in a 48-well plate in embryo water and TcdB_HIST_ or TcdB_HV_ across a range of concentrations was applied to the individual wells. At 24 h following treatment, a minimum of 20 zebrafish larvae per condition were examined by light microscopy for physiological changes, tissue damage, and viability (Fig. 2). Extensive necrosis was evident in all embryos exposed to TcdB_HV_, with broad tissue damage caused to the yolk sac, body, and head at concentrations as low as 1 nM (Fig. 2B and 2D). Furthermore, all zebrafish treated with TcdB_HV_ succumbed to the effects of the toxin within 48 h. In contrast, treatment with TcdB_HIST_ resulted in more specific damage at the cardiac region in approximately 75% of embryos, and was not immediately lethal (Fig. 2A). Incubation with higher doses of TcdB_HIST_ or for longer periods of time increased toxicity but did not alter the physiological damage from this toxin. These findings indicate that TcdB_HV_ impacts a broader number of cell types in vivo compared to TcdB_HIST_. However, corresponding to our previous report TcdB_HIST_ preferentially targets cardiac cells in the zebrafish embryo system.

**Figure 3:**
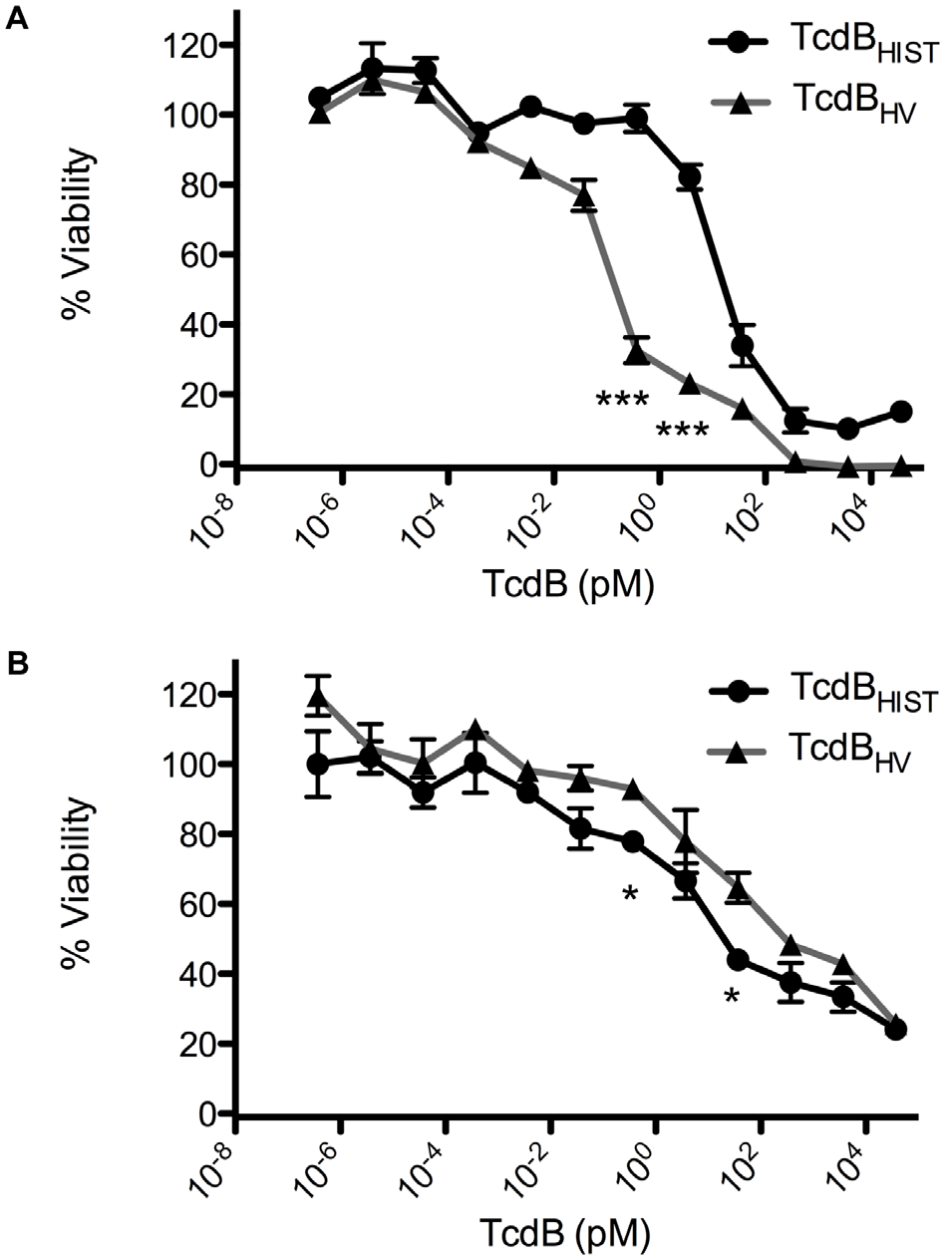
Comparative dose response of TcdB_HIST_ and TcdB_HV_. CHO or HL-1 cells were exposed to TcdB for 24 h and cell viability was determined by WST-8 staining. (A) TcdB_HIST_ (black) and TcdB_HV_ (gray) intoxication of CHO cells. (B) TcdB_HIST_ (black) and TcdB_HV_ (gray) intoxication of HL-1 cardiomyocyte cells. The error bars represent the standard deviation from the mean of three samples. * p<0.05, *** p<0.001.

Recent studies determined the relative cytotoxicity of TcdB_HV_ and TcdB_HIST_ on eight different cell types [17]. Because this analysis did not include cells of cardiac lineage, we compared the two toxins on HL-1 cells, which are derived from mouse cardiac tissue [38]. We also examined the effects of the two toxins on CHO cells for a relative comparison to the cardiomyocytes. As shown in Fig. 3, similar to previous observations, TcdB_HV_ was more cytotoxic to CHO cells (TCD_50_ 2.37×10^−13^ M) than was TcdB_HIST_ (TCD_50_ 2.53×10^−11^ M). In contrast, TcdB_HV_ was not more cytotoxic on cardiomyocytes and displayed a very similar activity to TcdB_HIST_. Upon further investigation of the cardiomyocytes, the cytotoxicity of TcdB_HV_ was found to be slightly lower than TcdB_HIST_ (p<0.05) with a TCD_50_ approximately 10-fold higher (3.37×10^−10^ M) than TcdB_HIST_ (TCD_50_ 2.80×10^−11^ M). These data indicate that while TcdB_HV_ has a broader cell tropism and is most likely more cytotoxic overall, TcdB_HIST_ cardiotropism is more pronounced between the two forms of this toxin.

### Comparison of intracellular effects of TcdB_HV_ and TcdB_HIST_

We next determined if the variation in cytotoxicity was due to differences in the cytosolic activities of the two forms of TcdB. As an approach to this problem we took advantage of a previously described system used for heterologous delivery of proteins and protein fragments into the cytosol of target cells [39], [40]. This system is composed of the cell entry components of anthrax lethal toxin. Briefly, protective antigen (PA) delivers lethal factor (LF) into the cytosol of mammalian cells. The heterologous delivery system is derived from the amino-terminus of LF (LFn), which interacts with PA and can be delivered into cells, but lacks enzymatic activity. In our experiments, the DNA fragment encoding the enzymatic domain of TcdB was genetically fused to *lfn*, yielding a DNA construct that expresses the cell entry portion of LF with the enzymatic component of TcdB. This heterologous delivery system allowed us to regulate the cell entry of the enzymatic component of TcdB_HV_ and TcdB_HIST_ so that these domains were identical in the way in which they entered the cell. We predicted that if the differences in cytotoxicity were due to factors other than intracellular activity of these forms of TcdB, then the fusions should exhibit identical cytotoxic effects.

**Figure 4:**
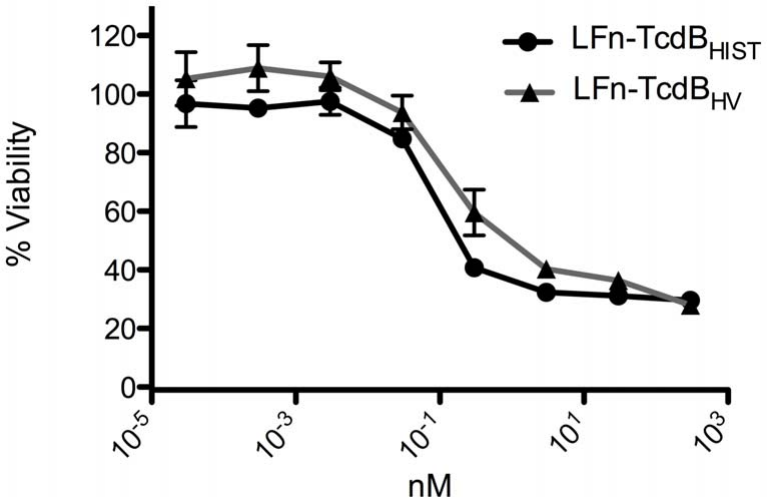
Heterologous delivery of the TcdB enzymatic domain. CHO cells were treated with LFnTcdB_HIST(enz)_ or LFnTcdB_HV(enz)_ in the presence of PA for 24 h and cell viability was determined by WST-8 staining. The error bars represent the standard deviation from the mean of three samples.

The results of the PA, LFn-TcdB fusion experiments are shown in Fig. 4. CHO cells were treated with a fixed amount of PA (500 nM) plus a range of concentrations of LFnTcdB_HV(enz)_ or LFnTcdB_HIST(enz)_ in order to generate a standard killing curve for this assay. As controls, CHO cells were treated with PA, LFnTcdB_HV(enz)_, or LFnTcdB_HIST(enz)_ separately. Following 24 h of treatment the cells were assayed for viability using WST-8 colorimetric assay and the percent survival was plotted versus concentration of the fusion protein. Treatment with each of the components alone had no effect on cell viability in this assay (data not shown). Treatments with PA plus LFnTcdB_HV(enz)_ or PA plus LFnTcdB_HIST(enz)_ resulted in similar (p<0.05) cytotoxicity at each of the concentrations tested (Fig. 4). To confirm that PA was not limiting in these experiments, cytotoxicity of the fusions was tested with 10-fold higher amounts of PA, and this additional amount of PA did not change the level of cytotoxicity for either fusion (data not shown). The results from this experiment suggested that the differences in the cytotoxicity of LFnTcdB_HV(enz)_ and LFnTcdB_HIST(enz)_ were not due to variations in intracellular activities of the enzymatic domains.

### Flow-cytometry analysis of TcdB_HIST_ and TcdB_HV_ interaction with CHO cells and cardiomyocytes

**Figure 5:**
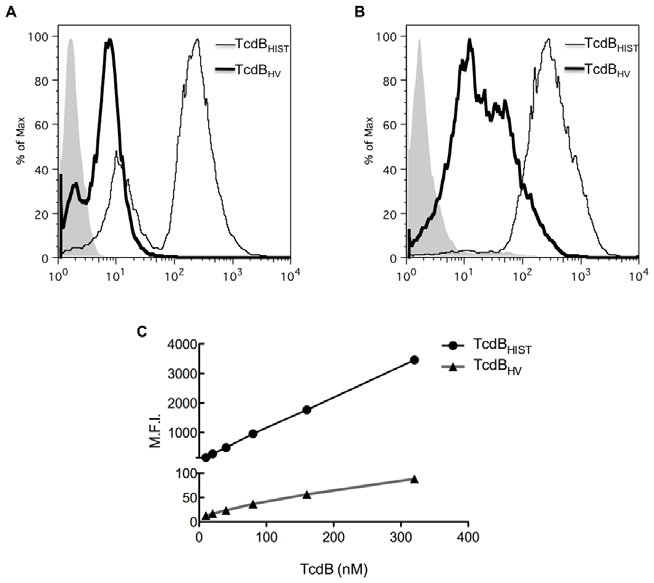
Flow-based analysis of cell binding. TcdB_HIST_ and TcdB_HV_ were tested for their ability to bind CHO (A) and HL-1 cells (B). 40 nM of fluorescently labeled TcdB was incubated with cells on ice, and binding was determined by flow cytometry. TcdB_HIST_ and TcdB_HV_ are indicated, and shaded peaks represent cells incubated with unlabeled TcdB. (C) Mean fluorescence intensity (MFI) vs TcdB concentration on HL-1 cells. Please note the difference in axis for TcdB_HIST_ and TcdB_HV_.

The results from the experiment using an identical method of cell entry, suggested the differences in cytotoxicity might be associated with early steps in cell binding and cell entry. To address this hypothesis, we compared the interaction of TcdB_HV_ and TcdB_HIST_ with cultured cells. Cultured cells were incubated with Alexa-647-labeled TcdB_HV_ or Alexa-647-labeled TcdB_HIST_ and the extent of toxin binding was determined by flow cytometry. This analysis was performed on CHO cell and HL-1 cardiomyocytes. As shown in Fig. 5, CHO cells and HL-1 cells exhibited a higher degree of fluorescence when incubated with labeled TcdB_HIST_ than when incubated with labeled TcdB_HV_. A biphasic profile was detected in CHO cells with a smaller population of cells exhibiting a distinct, reduced, toxin-binding pattern. In contrast, binding to cardiomyocytes was uniform and revealed a profile expected for a single population of cells.

Experiments were next performed to determine the apparent Kd for binding of TcdB_HIST_ and TcdB_HV_. Interestingly, within the constraints of these experimental conditions we were not able to achieve saturable binding of either form of the toxin to target cells. Fig. 5C shows a nearly linear correlation between the increase in toxin concentration and the mean fluorescence intensity (MFI) of HL-1 cells despite reaching toxin concentrations of over 300 nM. Additionally, Fig. 5C further emphasizes the extremely low level of interaction of TcdB_HV_ with target cells in comparison to the high MFI achieved with TcdB_HIST_. These data suggest that cell binding involves a higher order and more complex process than expected for a single receptor-ligand interaction.

### Rates of cell entry differ between TcdB_HIST_ and TcdB_HV_

**Figure 6:**
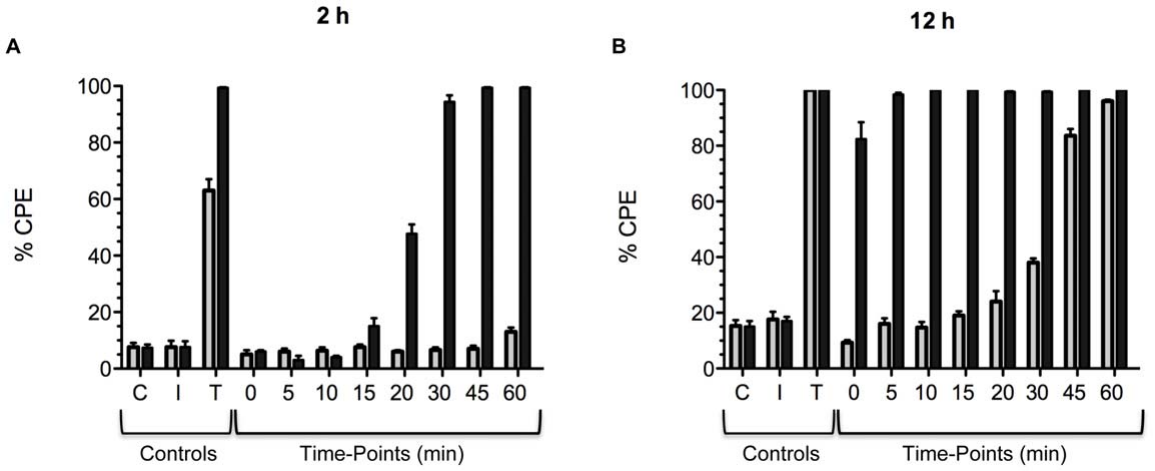
Comparison of the timing of cell entry between TcdB_HV_ and TcdB_HIST_. CHO cells were pretreated with TcdB_HIST_ or TcdB_HV_ and the lysosomotropic inhibitor, ammonium chloride, was added at the indicated time points. Cytopathic effects were determined at 2 h (A) and 12 h (B), and black bars represent cells treated with TcdB_HV_ while gray bars represent TcdB_HIST_. The error bars mark the standard deviation from the mean. C, untreated control. I, inhibitor alone. T, TcdB alone.

Experiments were next performed to assess the difference in the rates of cell entry between the two toxins. In previous work on historical TcdB, we found that lysosomotropic inhibitors could completely block cytopathic effects of the toxin for up to 16 h, even if added up to 20 min following exposure of the cells to the toxin [41]. These findings indicate interaction with the cell, uptake, and then translocation into the cytosol requires at least 20 min and acidification of endosomes is necessary. To determine if TcdB_HV_ differed from TcdB_HIST_ in rates of cell entry, cultured CHO cells were treated with the two forms of the toxin and a lysosomotropic agent was added to the cells at time-points ranging from 5 to 60 min following treatment with toxin. The lysosomotropic agent was also added prior to or at the same time cells were treated with the toxins. The effect of the lysosomotropic agent was then assessed by determining the level of cytopathic effects (CPE) either 2 h or 12 h after treatment with the toxin. For this experiment CPE was determined rather than cytotoxicity due to toxicity of ammonium chloride at the later time points necessary for cytotoxicity assays. As shown in Fig. 6, based on the extent of cell rounding, there appeared to be a clear difference in the rates of translocation between TcdB_HV_ and TcdB_HIST_. Unlike our earlier findings on TcdB_HIST_, the cytotoxic effects of TcdB_HV_ could not be prevented when the lysosomotropic agent was added as soon as 10 min following treatment with the toxin (Fig. 6A). Furthermore, addition of the lysosomotropic agent within 10 min of treatment of TcdB_HV_ only provided a slight delay in CPE, as all inhibitor treated cells showed complete rounding by 12 h (Fig. 6B). In contrast, the CPE of TcdB_HIST_ could be prevented by adding the inhibitor up to 30 min following treatment with the toxin. These findings indicate TcdB_HV_ translocates to the cytosol more rapidly than TcdB_HIST_.

### Hydrophobic transitions occur at a higher pH in TcdB_HV_

**Figure 7:**
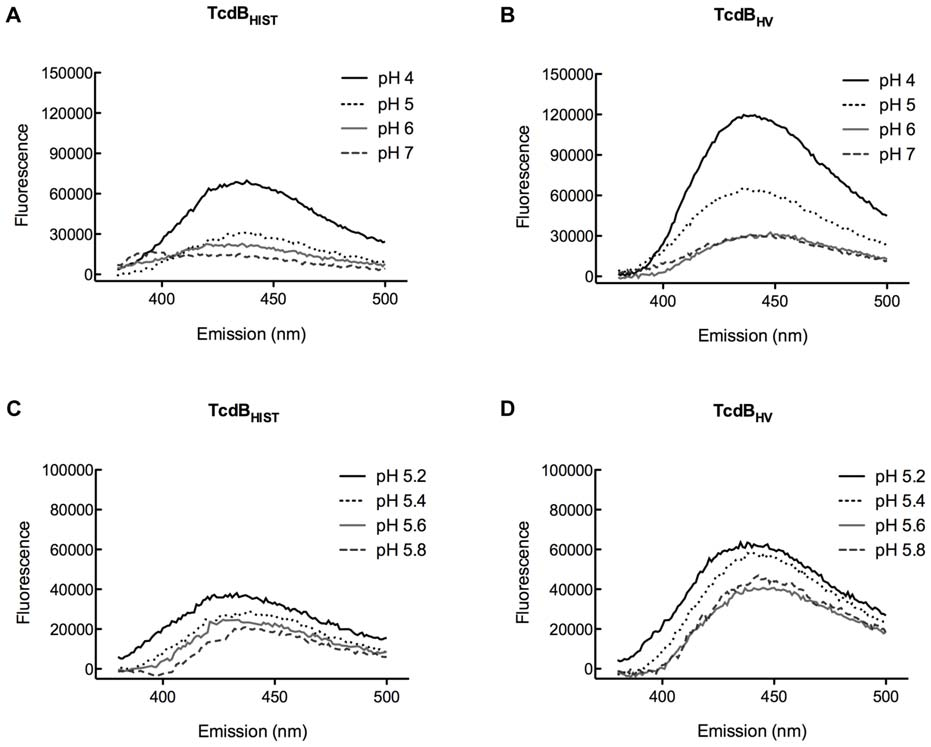
TNS analysis of pH-induced hydrophobic transitions in TcdB_HIST_ and TcdB_HV_. TcdB_HIST_ or TcdB_HV_ was incubated with TNS for 20 min at 37°C. Samples were analyzed for changes in TNS fluorescence, and the emission profile of each pH is shown and labeled. Panels (A) and (B) represent pH 4.0 to pH 7.0 and panels (C) and (D) show TNS fluorescence of TcdB between pH 5.0 and 6.0. Each spectrum represents the experimental sample with background (TNS and buffer alone) subtracted.

Previous studies from our group found that acidic pH triggers hydrophobic transitions in TcdB_HIST_ [41]. Studies by Barth et al. found that this hydrophobic transition in TcdB correlated with membrane insertion by the toxin [42]. These conformational changes corresponded to the decrease in endosome pH that led to translocation of the toxin into the cytosol. Thus, it was reasonable to suspect that TcdB_HV_ translocates more quickly into the cytosol because the hydrophobic transition was induced at a higher pH and thus at an earlier stage of endocytosis. To address this possibility, in the next series of experiments we identified the pH dependent conformational transitions of TcdB_HV_ by observing changes in TNS fluorescence when the toxin was incubated at various pHs. To identify whether TcdB_HV_ exhibits differential transitions compared to TcdB_HIST_, the proteins were preincubated with 150 µM TNS at pH 4.0, 5.0, 6.0, and 7.0, and then analyzed for changes in TNS fluorescence. As shown in Fig. 7, TcdB_HV_ exhibited a significant increase in hydrophobicity at pH 5.0, while TcdB_HIST_ did not undergo this transition until pH 4.0. Further examination of a narrower pH range revealed that a significant shift occurred between pH 5.4 and 5.6 in TcdB_HV_ (Fig. 7D). In comparison, TNS fluorescence of TcdB_HIST_ at these pHs was just above background levels.

**Figure 8:**
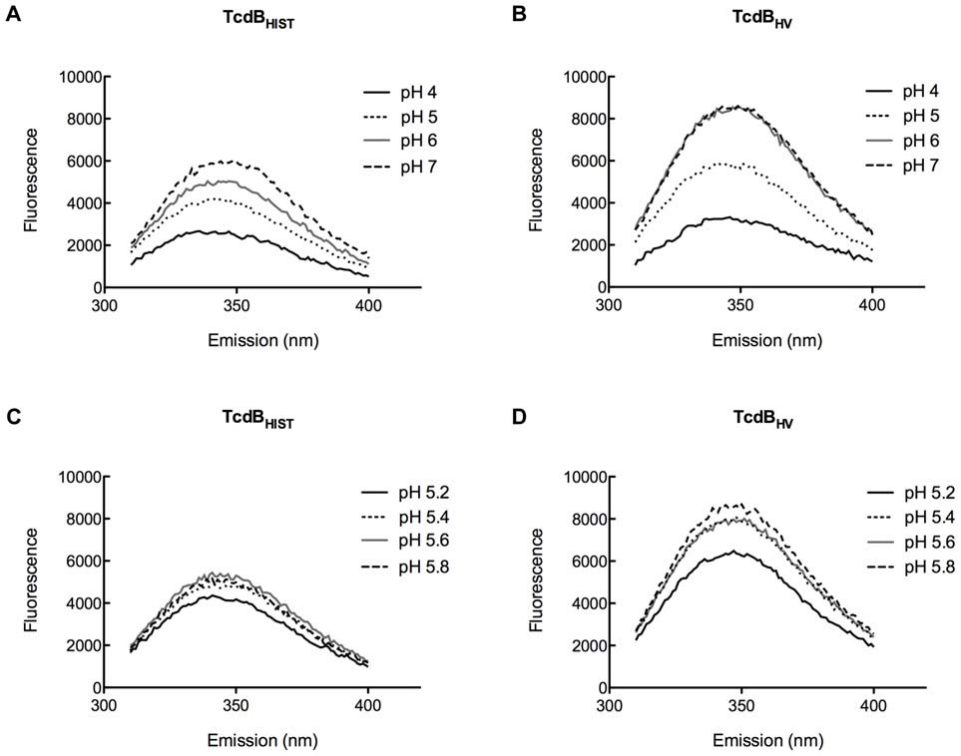
Tryptophan emission of TcdB_HIST_ and TcdB_HV_ at acidic and neutral pH. The fluorescent spectrum of each sample is shown and labeled; each spectrum represents the experimental sample minus background fluorescence of buffer alone. Panels (A) and (B) show tryptophan emission of TcdB_HIST_ and TcdB_HV_ from pH 4.0 to pH 7.0 while panels (C) and (D) highlight the changes in tryptophan fluorescence between pH 5.0 and pH 6.0.

These pH transitions were also studied using the inherent fluorescence of TcdB_HIST_ and TcdB_HV_ from the emission of tryptophan residues. Unfolding of the hydrophobic region should expose portions of the protein to a more aqueous environment, quenching tryptophan fluorescence. Environmental changes surrounding the tryptophan residues over a broad range of pH are shown in Fig. 8A and 8B. A gradual quenching of fluorescence was detected in TcdB_HIST_ from pH 7 to pH 4, while the tryptophan emission spectra of TcdB_HV_ indicated a sudden shift between pH 5 and pH 6. Fig. 8D reveals that this shift took place between pH 5.4 and 5.2, similar to the increase in TNS fluorescence seen at pH 5.4.

## Discussion

In the current study we compared the sequences and activities of TcdB from hypervirulent and historical strains of *C. difficile*. Because TcdB has been shown to be the major virulence factor of *C. difficile* [43], we reasoned that changes in the activity of this toxin could have a profound impact on the severity of disease. The findings support this notion, as TcdB_HV_ exhibited a broader tropism and higher potency than TcdB_HIST._ Among the possible explanations for this increased toxicity are the observations that TcdB_HV_ enters cells more rapidly than TcdB_HIST,_ and TcdB_HV_ undergoes conformational changes at a higher pH than TcdB_HIST._

Based on the sequence comparisons and the results of the experiments using the heterologous delivery system (Figs. 1 and 3), it appears that the differences in tropism and cytotoxicity are due to changes in regions outside of the enzymatic domain. Rapid cell entry could lead to more efficient cell killing by providing the toxin an endocytic condition in which the toxin is not subject to possible destruction by lysosomal proteases. The data from the lysosomotropic inhibitor assays (Fig. 6) support the idea that TcdB_HV_ does not reside within the endosome as long as TcdB_HIST_. Among the possible reasons for more rapid cell entry is a differential sensitivity to levels of IP_6_ that trigger autoproteolytic processing associated with translocation. We also noted a difference in the sequence of the hydrophobic region of TcdB, and if, as has been proposed [41], [42], this region mediates membrane insertion, such differences could allow TcdB_HV_ to insert into the membrane at an earlier stage of cell entry. We reasoned that if this possibility were true, there should be a difference in the pH-induced transitions of the two forms of TcdB, with the hydrophobic regions of TcdB_HV_ becoming exposed at a pH higher than the pH necessary for triggering this transition in TcdB_HIST_. The results from the TNS experiments (Fig. 7) indicate that TcdB_HV_ is able to undergo the hydrophobic transition at a higher pH than TcdB_HIST_, providing further evidence that TcdB_HV_ has higher translocation efficiency than TcdB_HIST_. Studies looking at the environment surrounding tryptophan residues of TcdB_HIST_ and TcdB_HV_ at lower pH (Fig. 8) support the idea that TcdB_HV_ undergoes a structural change at higher pH than TcdB_HIST_. Additionally, these experiments revealed that the transition of TcdB_HIST_ occurs gradually, while TcdB_HV_ demonstrates sudden shifts upon lowering the pH. This could be indicative of a more efficient unfolding of TcdB_HV,_ which may contribute to an enhanced ability to traverse the endosomal membrane. Our current working model is that TcdB_HV_ is able to translocate at an earlier point in endocytosis and this contributes, at least in part, to a more efficient intoxication.

We also recognize that the expanded tropism, along with more efficient cell entry could combine to enhance the in vivo toxicity of TcdB_HV_. The results from the zebrafish experiments (Fig. 2) indicate TcdB_HV_ targets a broader array of cells in vivo than does TcdB_HIST_. Defining the specific tropism in the murine model or an infection model is more difficult, but it is reasonable to consider the possibility that TcdB_HV_ is more lethal because the toxin targets an extensive variety of cell types systemically. Unfortunately, the TcdB receptor has been difficult to identify. Several attempts by our group to identify the TcdB receptor using standard techniques that have been successful with other toxins have failed. The results from the flow analyses in the current study suggest that the interaction of TcdB with the cell surface does not fit a single ligand-receptor model; this observation may explain why it has been so difficult to identify a receptor for this toxin. We were not able to achieve saturable binding, and interestingly TcdB_HV_ interacted less efficiently than TcdB_HIST,_ despite the fact that TcdB_HV_ is clearly more cytotoxic than TcdB_HIST_. Undoubtedly, future studies on characterizing this complex interaction with target cells will provide important insight into a novel mechanism of TcdB intoxication.

Previous work by Razaq et al. found that C. difficile BI/NAP1/027 strains were more lethal than historical strains of *C. difficile* [44]. As mentioned in the introduction of this paper, there are several differences in the phenotypes of the hypervirulent and historical strains of *C. difficile*. NAP1 strains sporulate at a higher efficiency and are resistant to fluoroquinolones. Both of these characteristics may make the NAP1 strains more difficult to manage in the hospital setting and increase the frequency of disease, but are unlikely to increase virulence. Likewise, the binary toxin has been shown to enhance colonization [13], but clinical data have revealed little correlation between the increase in disease severity and production of this toxin [45], [46]. In addition, previous work found binary toxin to be enterotoxic, but strains producing binary toxin alone did not cause disease in hamsters [47]. Clearly, an increase in toxin production such as that reported for NAP1 strains could enhance virulence, but a recent report suggests that the *tcdC* mutation in epidemic strains does not always correlate with the overexpression of TcdA and TcdB [48]. Based on the findings from the current study, we suggest that variations in TcdB sequence and activity could be an important determining factor in the hypervirulence of NAP1 strains.

The recent work of Lyras et al. [43] found that TcdB is critical to *C. difficile* virulence in a hamster model of CDAD. Thus, variations in the antigenic region (e.g. carboxy terminus) of TcdB could allow repeated *C. difficile* infections of the same host by strains with antigenic variants of this toxin. In a recent publication by He and colleagues it was estimated that *C. difficile* diverged into a distinct species between 1.1 and 85 million years ago, and has gone through remarkable genetic variation over time [49]. The authors also posited that immune selection could have influenced the genetic variation, and they examined candidate immunogenic proteins that might fit this profile and 12 such proteins were identified. TcdB was not among these candidate proteins. It is unclear whether TcdB fits the criteria established for a positively selected core gene of *C. difficile* in this study, but it is reasonable to suspect the gene may have varied to avoid immune responses and this hypervariability enriched for a more potent form of the toxin. It is worth noting that while the protein identity was around 92%, the DNA homology was 93%. Nearly all of the residue changes occur as a single nucleotide substitution that result in amino acid substitutions. This further suggests a possible change in the sequence of TcdB that has been selected through an enhancement in virulence and perhaps by immune evasion.

## Materials and Methods

### Reagents and cell culture

Chinese hamster ovary-K1 (CHO) cells were maintained in F-12K medium (American Tissue and Culture Collection; ATCC) along with 10% fetal bovine serum (ATCC). HL-1 cardiomyocytes were obtained from the Claycomb laboratory [38] and maintained in Claycomb medium (Sigma) supplemented with 10% fetal bovine serum (ATCC), 0.1 mM Norepinephrine (Sigma), and 2 mM L-glutamine (Invitrogen). Cultures were grown at 37°C in the presence of 6% CO_2_. *C. difficile* VPI 10463 (produces TcdB with identical sequence to the 630 strain) and *C. difficile* BI17 6493 (a gift from Dr. Dale Gerding), were used in this study for the purification of TcdB_HIST_ and TcdB_HV_. The *tcdB* gene was sequenced from both of these strains and the sequence was confirmed as exact matches to Genbank deposited sequences of strain 630 and R20291 (Genbank numbers AM180355 and FN545816). Cultures were grown as previously described [41], and TcdB was isolated by consecutive steps of anion-exchange (Q-Sepharose) and high-resolution anion-exchange (Mono-Q) chromatography in 20 mM Tris-HCl, 20 mM CaCl_2,_ pH 8.0. Purification steps were followed by protein determination using the Bradford method, visualization of a single band by SDS-PAGE, and LC/MS/MS analysis (University of Oklahoma Health Science Center) to confirm protein identity. Cytotoxicity was determined using a WST-8 [2-(2-methoxy-4-nitrophenyl)-3-(4-nitrophenyl)-5-(2,4-disulfophenyl)-2H-tetrazolium, monosodiumsalt] (Dojindo Laboratories) according to manufacturer's instructions.

### Zebrafish husbandry and experiments

Zebrafish maintenance and experiments were performed in accordance with the PHS Principles for The Utilization and Care of Vertebrate Animals Used in Testing, Research, and Training, and followed the recommendations in the Guide for the Care and Use of Laboratory Animals under the approval of The University of Oklahoma Health Sciences Center Campus IACUC (OUHSC #06-126). Zebrafish were obtained from Aquatic Eco-System (Apopka, FL). Zebrafish were maintained at 28.5°C on a 14 h light/10 h dark cycle in 10 gallon tanks equipped with pumps for mechanical and chemical filtration. Matings were performed in false bottom tanks, and embryos were washed briefly with 0.5% bleach after collection. Embryos were incubated in embryo water (60 mM NaCl, 1.2 mM NaHCO_3_, 0.9 mM CaCl_2_, 0.7 mM KCl) in petri dishes at 28.5°C, and water was changed daily. For TcdB treatment experiments, embryos were used between 48 and 72 h post fertilization, with chorions removed. Embryos were placed (5 embryos per well) into 48-well plates and treated with TcdB_HIST_ or TcdB_HV_ in embryo water at concentrations ranging from 50 nM to 0.01 nM. The embryos were observed for 72 h after treatment for morphological changes by using a SZX-7 microscope with a DP70 camera (Olympus). All images were captured and processed by using DP controller and DP manager software (Olympus).

### Construction of LFn-fusions and related assays

The region encoding the enzymatic domain of TcdB_HV_ was amplified from *C. difficile* NAP1 genomic DNA by PCR using the forward primer 5′-ACGTCCCGGGATGAGTTTAGTTAATA-3′ and the reverse primer 5′-ACTGGATCCTCATTATACTGTATTTTG-3′ to generate the *tcdB* gene fragment encoding residues 1 to 1668 of *tcdB* (*tcdB*_1–1668_) with a 3′ *Xma*I/*Sma*I and a 5′ *Bam*HI site. The restricted gene fragment was fused to *lfn* by overnight ligation at 16°C with a *Xma*1/*Bam*HI-restricted pET15b derivative containing *lfn*. The resulting plasmid was cloned into *Escherichia coli* XL-1 blue (Novagen) and candidate clones were screened for the correct insert and orientation by restriction analysis and DNA sequencing. LFnTcdB_HIST(enz)_ which had been previously cloned and described [40] and the newly synthesized LFnTcdB_HV(enz)_ were expressed using *E. coli* BL-21 Star (Invitrogen). Both fusions were purified by Ni^2+^ affinity chromatography (His-Trap, GE Life Sciences) and the purified protein migrated within the predicted size range of ∼94 kDa on SDS-PAGE. Protective antigen was expressed and purified as previously described [50].

### Cell binding analyses

TcdB_HIST_ or TcdB_HV_ were labeled with Alexa Fluor 647 C_5_ maleimide (Invitrogen) according to manufacturer's instructions. Briefly, a 10 M excess of dye was added to TcdB in 20 mM Tris-HCl, pH 8.0, and incubated overnight at 4°C. Conjugated protein was separated from unincorporated dye using Sephadex G-25, and efficiency of labeling was confirmed to be between 80% and 100%. The activity of labeled TcdB was confirmed by cytotoxicity on CHO and HL-1 cells and was not reduced by >10%. Binding of each toxin to CHO and HL-1 cells was examined as follows. Cells were dissociated from flasks using 1 mM EDTA in PBS, centrifuged at 500× *g*, and washed once with PBS. One hundred thousand cells were incubated with a range from 10 nM to 320 nM of labeled toxin in 1 mL of PBS on ice for 1 h, washed twice, and the pellets were resuspended in 1 mL of PBS. The samples were analyzed using a FACSCalibur flow cytometer (University of Oklahoma Health Sciences Center) and FLOWJO software (Tree Star, San Carlos, CA). The emission wavelength was set to 665 nm, and the excitation was set at 633 nm with a bandpass of 30 nm.

### Lysosomotropic inhibitor assays

CHO cells were plated at 5×10^4^ cells/well in a 96-well plate and incubated overnight. The following day, TcdB_HIST_ or TcdB_HV_ was added to the cells at a final concentration of 0.1 µg/mL. At the indicated time points, the cells were washed to remove unbound toxin, and ammonium chloride (Sigma) was added to the cells at final concentration of 100 mM. Each sample was monitored for 24 h, and CPE (cytopathic effect) was determined by visualization. Percent CPE was calculated by counting a minimum of 100 cells in 3 different fields for each sample. Cells scored positive for CPE only when fully rounded, and the percent CPE was calculated as % rounded cells_test_ - % rounded cells_control_, where control refers to cells treated with media alone.

### TNS assays and tryptophan analysis

2-(*p*-Toluidinyl) naphthalene-6-sulfonic acid, sodium salt (TNS; Invitrogen) solutions were prepared to a final concentration of 150 µM in pH specific buffers. For pHs ranging from 4.0 to 6.0, 100 mM NaCl-100 mM ammonium acetate-1 mM EDTA was used. For pH 6.0 to 7.0, 100 mM NaCl-100 mM MES-1 mM EDTA was used. For pH 7.0 to 8.0, 100 mM NaCl- 100 mM HEPES-1mM EDTA was used. 40 pmol of TcdB_HIST_ or TcdB_HV_ was added to the buffer/TNS mixture in a final volume of 2.5 mL and allowed to incubate for 20 min and 37°C. Each sample was analyzed on a Fluorolog R928P PMT fluorometer (HORIBA Jobin Yvon) with an excitation of 366 nm and an emission scan of 380 to 500 nm with a slit width of 2.0. Tryptophan fluorescence of TcdB_HIST_ and TcdB_HV_ was also compared in the same manner, using an excitation of 270 nm and an emission scan of 310 nm to 400 nm.

### Statistical analyses

Data are expressed as the means ± S.E.M. Statistical analyses were performed using two-tailed unpaired Student's t-test in GraphPad Prism (La Jolla, CA). Statistical significance is indicated as * p<0.05; ** p<0.01; *** p<0.001.
